# *R*-Loci Arrangement Versus Downy and Powdery Mildew Resistance Level: A *Vitis* Hybrid Survey

**DOI:** 10.3390/ijms20143526

**Published:** 2019-07-18

**Authors:** Elena Zini, Chiara Dolzani, Marco Stefanini, Verena Gratl, Paola Bettinelli, Daniela Nicolini, Giulia Betta, Cinzia Dorigatti, Riccardo Velasco, Thomas Letschka, Silvia Vezzulli

**Affiliations:** 1 Laimburg Research Centre, Laimburg 6, 39052 Vadena (BZ), Italy; 2 Research and Innovation Centre, Fondazione Edmund Mach, via E. Mach 1, 38010 San Michele all’Adige (TN), Italy; 3 Institute of Pharmacy/Pharmacognosy, Center for Molecular Biosciences Innsbruck (CMBI), University of Innsbruck, Innrain 80/82, 6020 Innsbruck, Austria; 4 CREA Research Centre for Viticulture and Enology, Via XXVIII Aprile 26, 31015 Conegliano (TV), Italy

**Keywords:** disease resistance loci, *Erysiphe necator*, grapevine, marker-assisted breeding, pyramiding, *Plasmopara viticola*

## Abstract

For the viticulture of the future, it will be an essential prerequisite to manage grapevine diseases with fewer chemical inputs. The development and the deployment of novel mildew resistant varieties are considered one of the most promising strategies towards a sustainable viticulture. In this regard, a collection of 102 accessions derived from crossing *Vitis* hybrids with *V. vinifera* varieties was studied. In addition to the true-to-type analysis, an exhaustive genetic characterization was carried out at the 11 reliable mildew resistance (*R*) loci available in the literature to date. Our findings highlight the pyramiding of *R*-loci against downy mildew in 15.7% and against powdery mildew in 39.2% of the total accessions. The genetic analysis was coupled with a three-year evaluation of disease symptoms in an untreated field in order to assess the impact of the *R*-loci arrangement on the disease resistance degree at leaf and bunch level. Overall, our results strongly suggest that *R*-loci pyramiding does not necessarily mean to increase the overall disease resistance, but it guarantees the presence of further barriers in case of pathogens overcoming the first. Moreover, our survey allows the discovery of new mildew resistance sources useful for novel QTL identifications towards marker-assisted breeding.

## 1. Introduction

Plant diseases cause billions of dollars in lost harvest annually, and in some instances, these losses have severe consequences for humans. One of the most convenient, inexpensive and environmentally sound ways to control plant disease is to utilize disease-resistant varieties, and plant breeders make extensive use of classically defined *R* genes [1]. Recent work has revealed the structure of a number of plant *R* genes, and a striking degree of similarity among these genes has been observed. The majority of resistance genes encode proteins classified as NBS-LRR proteins because they contain a nucleotide binding site (NBS) domain and a leucine–rich repeat (LRR) domain, followed by Toll/Interleukin-1-receptors (TIR); Coiled coil (CC); Transmembrane domain (TrD); PEST aminoacid domain; Endocytosis cell signaling domain (ECS); Nuclear localization signal (NLS); WRKY amino acid domain; Helminthosporium carbonum (HC) toxin reductase enzyme. Some categories can be more frequently associated with specific resistances (e.g., fungi vs oomycetes) [2]. Many resistance genes occur in complex loci that contain multiple copies of closely-related gene sequences. The concept of haplotype is used to describe the precise complement of related genes occurring in a particular variant of a complex locus. By contrast, there are some simple loci that encode only a single known resistance allele [3].

In grapevine, up to now a list of 27 genomic regions is reported associated with downy mildew (DM) resistance and 13 related to powdery mildew (PM) resistance [4], hereafter referred as disease resistance (*R*) loci. So far the thorough information about responsible genes has been achieved through map-based cloning approaches in a few cases: *Run1* [5,6], *Rdv1* [7] and *Rpv1* [8]. The genetic base of resistance to PM, DM and other grapevine diseases originates from *Vitis* species which are natural sources of resistance, mainly deriving from North America and more recently from the Far East [9]. Most of them confer varying levels of partial resistance, while others coming from *Muscadinia rotundifolia* and *V. piasezkii* confer total resistance in the genetic context where they are studied. The disease resistance breeding history in grapevine began in the late 19th century, when the import of grapevine material from America into Europe (primarily France) enabled the introduction of serious, previously absent grapevine pests and pathogens for which the native European vines (*V. vinifera* L.) had no resistance. In particular, the North American genotypes were used for the reason of being resistant to phylloxera and the first interspecific hybrids, mainly rootstocks, were bred to overcome the insect threat [10]. Starting in the second half of the 19th century, several attempts on combining different resistance traits of American grapevines (*V. riparia, V. labrusca, V. aestivalis* and *V. berlandieri*) with qualitative characteristics of European species were made, leading to the creation of interspecific resistant varieties. Besides the phylloxera plague, a serious invasion of fungal diseases contributed to the massive destruction of European vineyards and led to the increase of hybrids. Very important in the history of interspecific breeding are the genotypes coming from France called first-generation hybrids, usually meaning crossings between American species and cultivated French varieties, also called “direct producers”, i.e., grapes grown on own roots and used for wine making [10]. This kind of crossings was made in the first quarter of the 20th century mainly by the breeder Albert Seibel. The second-generation hybrids, basically crossings between first generation hybrids among themselves or with cultivated European varieties, came out later in the century and were performed by Bertille Seyve and Victor Villard. They contain a higher percentage of the *vinifera* genome, thereby increasing the quality of the wine [11]. More recently, marker-assisted selection in combination with several backcrossing with *vinifera* varieties led to the development of fungi resistant grapes carrying multiple disease resistance genes and a significant percentage (more than 85%) of *vinifera* in their pedigree [12,13].

Despite a European Council Regulation (No 1493) in 1999 [14] that enabled to produce “quality wines” only from varieties belonging to botanical species *V. vinifera*, resistant cultivars are frequently used in some northern European viticultural regions. Environmental, health and cost concerns are leading producers to reconsider hybrids or interspecific crossings. First and foremost is the worry about the need for intensive use of fungicides to control diseases. A 2003 report of the European Food Safety Authority (EFSA) estimated that viticulture uses 40% of the crop protection products in European agriculture. For this reason, the future trend is addressed to reduce pesticides as much as possible [15]. In this respect, the almost 100 years of breeding efforts for interspecific crossings cannot be considered as unnecessary. Eventually, the attitude is changing: In a 2009 revision [16], the European Union relaxed the regulations prohibiting hybrids. Although the highest classification (Protected Designation of Origin, PDO) requires *V. vinifera* varieties, hybrids can be used in the next level (called Protected Geographical Indication, PGI). Interspecific, disease resistant hybrids are generally referred to as PIWI (from German: pilzwiderstandsfähig, meaning “fungal disease resistant”) and they are now accepted as *V. vinifera* varieties in the most European Catalogues [17]. Nowadays “PIWI” is also the name of a producer group devoted to the “dissemination of fungus-resistant grape varieties” with 350 members from 17 European and North American countries, some of whom running private breeding programs [18]. Moreover, in order to sustain the resistant variety growth in the marketplace, producers will need to overcome the stigma still associated with hybrid-derived wines. 

Nowadays, on the one hand, the situation seems very favourable for the genetic improvement for disease resistance in grapevines: Many resistance sources are available, and several resistance factors with various effects have already been discovered. On the other hand, we know that disease resistances are not necessarily stable traits, and the protection could be “quickly” overcome by a virulent strain of the pathogen [19]. The advent of molecular genetics and associated technology like marker-assisted selection has led to the emergence of a new field in plant breeding, gene pyramiding [20]. It is now widely accepted that pyramiding resistance genes could be an effective plan to control a large range of pathogen strains as well as combining various defence mechanisms, all valuable strategies to increase resistance durability. Unfortunately, durability—the preservation of disease resistance genes over time—also depends on environmental conditions and agronomic practices, which influence the development of pathogen populations. From a practical point of view, the sustainable management of resistance aims at reducing the selection pressure applied by the resistance genes on the pathogen populations thanks to potentially durable genetic constructions and resistance deployment strategies, including cultivation practices [21]. One of the missing links for further improvement in breeding is a phenotypic evaluation of the genetic resources in the same environmental conditions taking into account the presence of resistance loci pyramids in their genome.

The aim of this study was to fill this gap assessing DM and PM resistance symptoms in a set of 102 accessions grown in an untreated field located in Northern Italy. The three-year experiment allowed the evaluation of the genetic material’s response to DM and PM in field compared to the *R*-loci arrangement in every accession and could gather the effect of different *R*-loci, alone or in pyramids, on mildew disease outbreaks in the same environmental conditions. 

## 2. Results and Discussion

### 2.1. Fingerprinting

**Figure 1:**
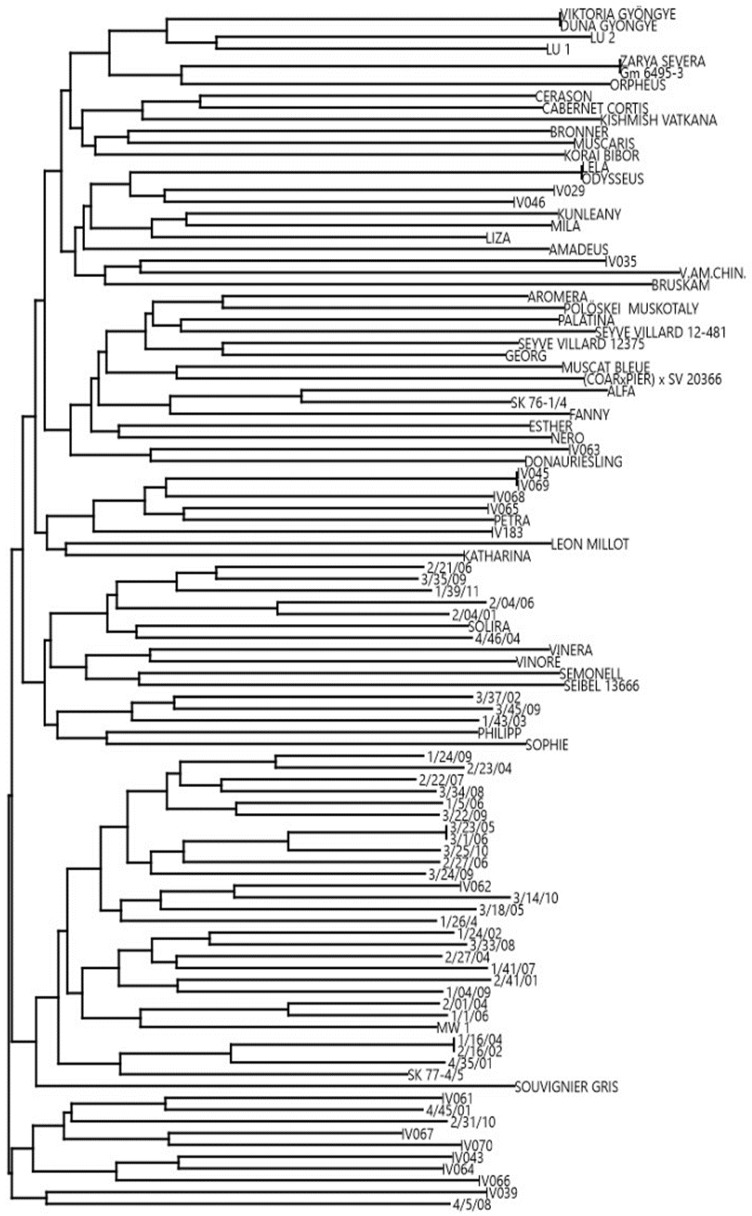
Dendrogram showing the genetic distance based on cluster analysis (Neighbour Joining clustering; Euclidean similarity).

The present collection was studied within the international, collaborative project VITISANA and is thus referred to as the VITISANA collection in this work. It comprises 102 accessions, 18 of which were confirmed as true-to-type (TTT) by DNA profiling and comparing profiles at the Vitis International Variety Catalogue (VIVC) database [9]. Twenty-five profiles corresponded to well-known accessions without genetic information available at VIVC database. For four accessions (‘Duna Gyöngye’, ‘Odysseus’, ‘Viktoria Gyöngye’ and ‘Zarya Severa’) the TTT was not validated since they showed another genetic profile according to the VIVC database. On a total of 55 accessions, including 37 progeny individuals and 18 breeding lines, TTT results could not be disclosed since they are part of selections by private breeders (data on pedigree not available) (Table S1). For this reason, to increase the genetic information, a dendrogram reporting the genetic distance among all studied accessions is depicted in Figure 1. The dendrogram showed five pair identities (‘3/23/08’ = ‘3/1/06’; ‘Zarya Severa’ = ‘GM6495-3’; ‘Viktoria gyöngye’ = ‘Duna Gyöngye’; ‘Lela’ = ‘Odysseus’, ‘IV045’ = ‘IV069’), revealing the presence of 97 unique genetic profiles (genotypes). Taking into consideration the origin of the genotypes, we noticed a cluster comprising most of the progeny individuals in the first node located at the lower part of Figure 1. We speculated that this group shared at least one parent, thus identified as the candidate MW1 or IV062 genotypes based on 9 (highly polymorphic and neutral) reference SSR data.

### 2.2. R-Loci Characterization

In an “all-vs-all” approach, all *Vitis* hybrids were examined at the 11 screenable and reliable *R*-loci highlighting the presence of eight *R*-loci (*Rpv1*, *Rpv3*, *Rpv10* and *Rpv12* for DM and *Run1*, *Ren1*, *Ren3*, *Ren9* for PM) in a single (e.g., *Ren1*), combined (e.g., *Rpv12* + *Ren9*) and stacked/pyramided (e.g., *Rpv10* + *Rpv3-3*) status. The latter definition is in agreement with [22]. The *Rpv3* locus represents a peculiar case where different resistant haplotypes were characterized [23] and for which we referred to a paired status (e.g., *Rpv3-1* + *Rpv3-2*). The absence of both *Run2.1/2.2* variants as well as *Rpv14* and *Ren2* underlined the respective lack of cultivars derived from *M. rotundifolia* and of *V. cinerea* accessions in the pedigree of the analyzed genotypes. Certain *R*-loci were not taken into consideration from the beginning since their donor was private and not exploitable to develop any accessions belonging to the VITISANA collection; this is the case of *V. romanetii* (*Ren4*, [24,25]) and *V. piasezkii* (*Ren6* and *Ren7*, *Rpv15* and *Rpv16*, [26]; Pap et al., in preparation). Not for all *R*-loci, an appropriate choice of markers could be retrieved from literature. Although we have taken into consideration the exhaustive overview of traits and original donor variety or species accession available at [9], we identified only for some *R*-loci associated with DM and PM resistance a defined set of associated SSR markers that were robust and exploitable for Marker-Assisted Parental Selection (MAPS) and the derived Marker-Assisted Seedling Selection (MASS) practice. Reasons for this are (i) the lack of clear information, especially in the oldest publications, (ii) large genomic intervals that would require additional fine mapping, and (iii) the absence of confirmed QTLs that would request marker validation in additional populations. In fact, moving from publication to application domain is challenging. Especially for fruit trees, most of the publication results derive from investments from funders with a strategic scientific mission, leading to rare emphasis on applied value in breeding programs.

As stable and co-dominant markers, SSRs are currently the marker system of choice for the Marker-Assisted Breeding (MAB) program in grapevine, where reliable, efficient and cost-effective molecular markers have to be available. This type of markers demonstrated to provide robust phenotype correlation with disease resistance (e.g., [22]) as well as other traits. Indeed, SSRs have some limitations, such as the need of an expensive equipment for allele sizing through capillary electrophoresis and high mutation rates compared to other DNA markers. The latter tendency generates size variation in DNA regions otherwise identical-by-descent and by their model of evolution, which vice versa produce identical electromorphs via independent mutational events—known as homoplasy—confounding the studies of genetic variation within and among populations (reviewed by [27]). From here comes the need of developing new flanking markers and to convert the original ones into less variable marker types, as the point mutation-based. To date, although few SNPs – also in terms of haplotype blocks – have been developed for grapevine Marker-Assisted Selection (MAS) applications (e.g., [28,29]), they are becoming more favoured as a marker system, since they are amenable to high-throughput genotyping platform [30]. Lately, to bridge the gap between marker development and MAS implementation, a novel practical strategy with a semi-automated pipeline, which incorporates trait associated SNP discovery, low-cost genotyping through amplicon sequencing and decision making, has been developed [31]. Unlike microsatellites and more similar to point mutations, each InDel is a unique and irreversible molecular event, which helps tagging more effectively a given haplotype. For this reason, [27] have very recently discovered InDel tags for the *Rpv3-1* haplotype and proposed them as a significant improvement in terms of marker informative content, ease of allele scoring and MAS efficiency.

A list of the *R*-loci detected in each genotype of the VITISANA collection is shown in Table 1. Considering the incidence of each *R*-locus independently from its combination with other *R*-loci, a prevalence of *Rpv3, Ren3* and *Ren9* was observed. Regarding *Rpv3*-dependent resistance to DM, the *Rpv3-1* haplotype was the most frequent (56.9%, deriving from ‘Seibel 4614′), while the *Rpv3-2* (6.8%, conserved in the ‘Munson’ lineage) and the *Rpv3-3* haplotype (3.9%, tracing back to ‘Noah’) were fewer. The *Rpv3-1* haplotype was firstly identified in the German hybrid ‘Regent’ [32,33] and in the Hungarian hybrid ‘Bianca’ [34] through QTL mapping. The *Rpv3-1* presence originates from ‘Seibel 6468′, the only offspring of the ancestor ‘Seibel 4614′ that disseminated extensively the haplotype in the germplasm repositories until now. ‘Seibel 6468′ participated in the generation of ‘Villard blanc’ (also known as ‘Seyve Villard 12-375′), one of the most deployed hybrids in grapevine breeding programs. The predominance of this haplotype definitely indicates its fixation during selection since it confers a superior resistance with an ETI (effector triggered immunity) associated necrosis perfectly capable to restrict the pathogen [27]. *Rpv3-1* resistance depends on an inducible response specifically elicited by an avirulent strain of *P. viticola* and is a typical Hypertensive Response (HR), compatible with the cascade of events initiated by the products of NB-LRR and receptor like protein kinase genes, located within the *Rpv3-1* locus [35]. Upon the comprehensive study reported by [23], two further wild relative *Rpv3* haplotypes have been validated in segregating populations: *Rpv3-2* has recently been confirmed by QTL mapping in ‘GF.GA-47-42′ × ‘Villard Blanc’ segregating population [36], while *Rpv3-3* has been characterized in a ‘Merzling’ × ‘Teroldego’ progeny [37]. These two haplotypes are less represented in grapevine breeding selections [23] and therefore are novel and valid allelic variants, considering that *Rpv3-1* was discovered to be ineffective against a specific *P. viticola* isolate [19,38].

The second *R*-locus against DM well represented in the VITISANA collection was *Rpv12* (17.3%), coming from ‘Zarya Severa’ and deriving from *V. amurensis*. The Amur grape is native to the cool climate of the Far East (Siberia, China, Korea and Japan) arousing the interest of breeders concerned to incorporate cold tolerance into *V. vinifera* (e.g., expedition by Vavilov in 1920–1940). Moreover, they noticed that some accessions were not significantly damaged by *P. viticola* under conditions highly conductive to DM. Soviet breeders (Michurin, Negrul and Potapenko) contributed to the introduction of these accessions into the Russian breeding program, and thanks to the networking with the Soviet bloc, Eastern European breeders shared Amur material that since 1960s was present in the Continental Europe [39]. Before *Rpv12*, another *V. amurensis* resistance gene, *Rpv10* coming from ‘Severnyi’ [40], a full sibling of ‘Zarya Severa’, was discovered. In the VITISANA collection *Rpv10* is present with a percentual of 7.8%. For both *Rpv10* and *Rpv12*, only QTL mappings are available in the literature to date, while lacking gene expression or functionals studies. For both loci, the presence of large CC-NBS-LRR clusters has been detected along the reference genome [39,40].

Concerning the resistance to PM, *Ren3* (50%) and *Ren9* (49%) (both derived from ‘Regent’) were the most abundant, followed by a few genotypes with *Run1* (8%, derived from *M. rotundifolia*) and only one genotypes with *Ren1* (coming from *V. vinifera* cv. Kishmish vatkana). *Run1* (and *Run2*), as an example, derive from *M. rotundifolia*. Although introduced in Europe in the late 19th century together with most of the other American *Vitis* species, it has not elicited any real interest in European growers, since all of the few cultivation attempts failed at that time [41]. Only a pseudo-backcross strategy succeeded in the introduction of the single locus in the *V. vinifera* genome [42], allowing to use it for resistance breeding programs. Also *Ren1* from *V. vinifera* cv. Kishmish vatkana [43] was only recently utilized for pyramiding multiple resistances. Both *Ren3* [32,33,44,45] and *Ren9* [46] are located on chromosome 15 and are frequently inherited together. Being firstly discovered in ‘Regent’, the two *R*-loci originate from ‘Chambourcin’, one of the resistant varieties coming from French breeding efforts [23] and a popular parental line for breeders. This is probably the reason why, compared to the other PM resistance traits, these loci are quite widespread among the VITISANA genotypes. All the major PM responsive QTLs known as *Run/Ren* loci have been mapped in positions where various RGAs encoding TIR-NBS-LRR and CC-NBS-LRR type resistance proteins where mapped [47].

Interestingly, 12.7% and 33.3% of the genotypes were missing any of the analysed *R*-loci associated with DM and PM resistance, respectively. This suggests that these genotypes either are susceptible to one of the two diseases or represent new sources of resistance against DM or PM. The fact that most genotypes lack PM resistance demonstrates that breeding activities, concentrated in temperate-humid climates, are preferably focused on introducing resistance to DM.

**Figure 2:**
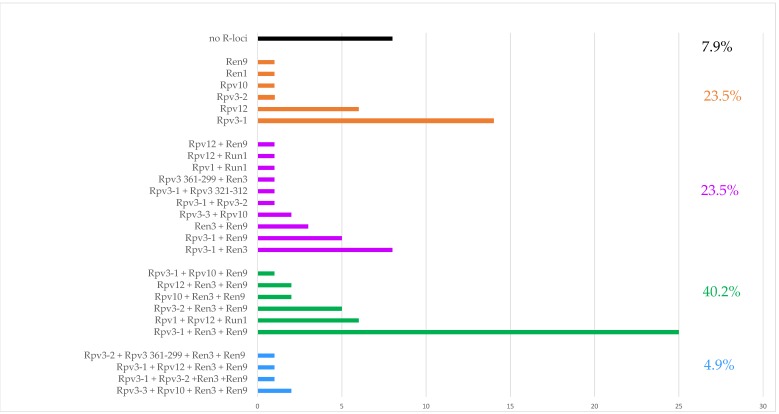
Grouped bar chart showing the percentual of accessions with single resistance locus, combination of two, three and four resistance (*R*) loci and absence of any studied *R*-locus. *Rpv*: Resistance to *Plasmopara viticola*; *Run*: Resistance to *Uncinula necator* (from *Muscadinia* spp.); *Ren*: Resistance to *Erysiphe necator* (from *Vitis* spp.).

Considering the total number of *R*-loci detected in each genotype, a molecular picture of the VITISANA collection is shown in Figure 2. Within the collection, 23.5% of the genotypes carried a single *R*-locus associated with DM or PM resistance, and the same percentage carried two loci. Further, our findings showed that pyramiding (stacking of at least two *R*-loci against the same disease) of *Rpv* loci occurred in 15.7% and of *Run*/*Ren* loci in 39.2% of the collection (data not shown). A particular case is represented by the paired status *Rpv3-1* + *Rpv3-2* detected in ‘MW1’ and ‘Katharina’ at the hypervariable *Rpv3* locus. In addition, 40.2% contained three loci with the combination 2 *Rpv* loci + 1 *Ren*/*Run* locus or 1 *Rpv* locus + 2 *Run*/*Ren* loci. Only 4.9% contain four *R*-loci (2 *Rpv* + 2 *Run*/*Ren* loci) and therefore resulted pyramided for both diseases. In 7.9% of the genotypes, no *Rpv* and *Run*/*Ren* locus was found at all according to analyses with the markers able to reliably foresee resistance traits; these selections represent putative novel sources of disease resistance, prior the consideration of their phenotypic data. Taking into account the dendrogram (Figure 1) and considering the *R*-loci arrangement, we observed a group (from ‘Lela’ to ‘Liza’ in Figure 1) carrying *Rpv12*, followed by a cluster (from ‘IV045′ to ‘Petra’) with *Rpv12* in some cases combined with *Run1*. We also identified a group from ‘IV067’ to ‘IV066’ characterized by *Rpv1* + *Rpv12* + *Run1*; these genotypes belong to the same breeding objectives of the private breeding platform InnoVitis based on closely related resistance sources (Tutzer E., personal communication). As expected, progeny individuals carrying *Rpv3-1* + *Ren3* + *Ren9* clustered together.

These results provided an overview on the genetic material present in the VITISANA collection. Putting them in relation with other data is challenging since few articles describe the presence of resistance QTLs for DM and PM in genotypes derived from breeding programs (e.g., [22,25,47,48]). The literature rather tends to concentrate on QTL studies and association analyses in order to deepen the knowledge on *R*-loci, as well as reported reviews on process [12] and dissertations on perspectives, especially in the post-genomics area [49,50]. On the contrary, data on MAB activities traditionally are not reported. In fact, as mentioned above about QTL validation, translating the research findings into application cases is mostly considered as not relevant and not required. In addition, the breeders’ mentality consists of considering the *R*-loci characterization as a simple, although advanced, tool in order to reach quickly the goals of yield and quality grape. 

Worldwide schemes for pyramiding resistance QTLs are currently applied in breeding programs for wine grapes, table grapes and rootstocks to boost cultivar development via MAS, including early seedling selection and parental choice prior to crossing, all focused on QTLs with major effect and none on QTLs with minor effects. The shared idea was to combine resistance QTLs with complementary modes of action for breeding effective and potentially durable resistance, relying on the fact that the effects of resistance QTLs are often additive. In addition, another relevant factor to be taken into account is that, if the target QTL contains resistance genes and their homologs tightly linked to genes with large negative effects on other traits, these undesirable genes maybe transferred together with the target gene into the recipient line and result in the reduced performance of other traits (linkage drag) [20]. Finally, given the relatively recent history of pyramiding in grapevine, the last element to consider is the (future) genetic load. The average individual taken from a population with a low genetic load will generally, when grown in the same conditions, have more surviving offspring than the average individual from a population with a high genetic load. Deleterious mutation load is the main contributing factor to genetic load overall. Inbreeding increases homozygosity. In the short run, an increase in inbreeding increases the probability with which offspring get two copies of a recessive deleterious alleles, lowering fitness via inbreeding depression. However, in a species that habitually inbreeds, e.g., through self-fertilization, recessive deleterious alleles are purged [51]. Since grapevine breeding plans in case foresee one step of self-crossing, the possibility to purge deleterious alleles is remote. Another contributor to the genetic load overall is the recombination/segregation load. Combinations of alleles that have evolved to work well together may not work when recombined with a different suite of coevolved alleles, leading to outbreeding depression. Segregation load is the presence of underdominant heterozygotes (i.e., heterozygotes that are less fit than either homozygote). Recombination load arises through unfavourable combinations across multiple loci that appear when favourable linkage disequilibria are broken down. Recombination load can also arise by combining deleterious alleles subject to synergistic epistasis, i.e., whose damage in combination is greater than that predicted from considering them in isolation [52]. In the case of mainly outcrossed species as grapevine, these events can definitely occur.

### 2.3. DM and PM Resistance Evaluation in an Untreated Field

**Figure 3:**
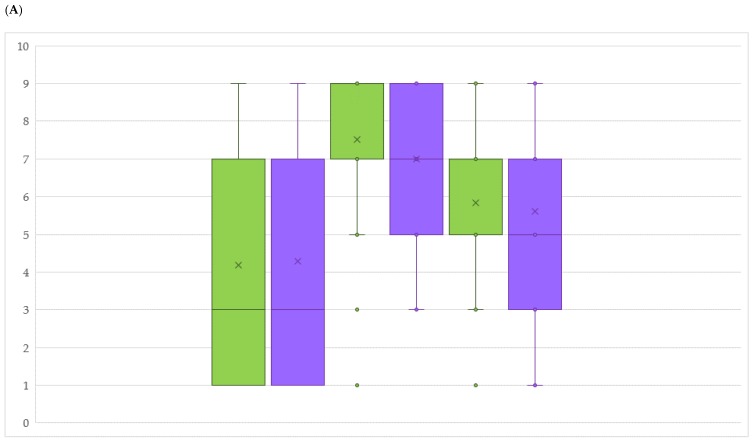

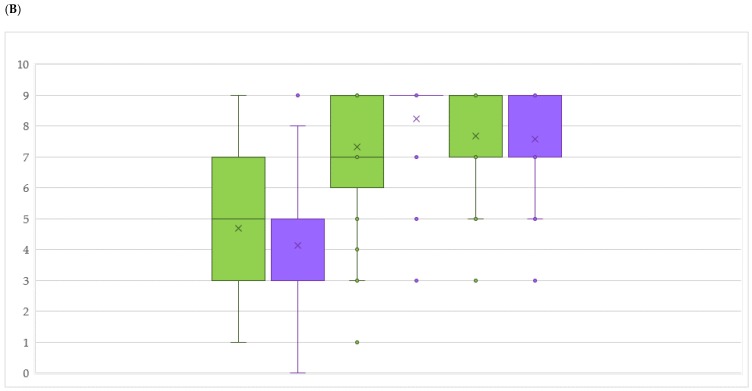
Box plots comparing OIV scores of resistance level in years 2016, 2017 and 2018 for downy (**A**) and powdery (**B**) mildew on leaves (green) and bunches (violet). Per each year, the lowest score between evaluations was chosen.

Even though the collection consists of 102 accessions, only 89 were available for field disease evaluations. The reason is attributable to low yields for 13 genotypes, impeding DM and PM bunch assessment in both periods during the first two years. Comparing the distribution of the genotypes according to standard scores defined by the International Organisation of Vine and Wine (OIV) and describing DM resistance level on leaves and bunches, for both organs we noticed a clear difference between the three years. In 2016, resistance levels on leaves and bunches ranged from low to mid degree, whereas in the two following years, the distributions significantly skewed towards a high (2017) and a moderate level of resistance (2018) (Figure 3A,B, Figure S1, Table S2). PM resistance was observed to be significantly lower in 2016, while increasing in 2017 and 2018, with 2016 remaining the most susceptible year in both disease observations.

Weather patterns revealed that 2016 was the rainiest year with a seasonal rainfall of 522.4 mm, whereas in 2017 and 2018 the rainfall in the same period was 491.4 mm and 381.2 mm, respectively (Figure S2). Interestingly, in 2016 the rainfall was particularly concentrated in April and May (sum of 183.4 mm), whereas it reached only 91 mm and 127 mm, respectively, in the two successive years. Indeed, the differences in DM resistance between the years can be attributed to the varying weather conditions, especially to rainfall [53]. Rain can initiate the process of oospore germination, breaking the dormancy (primary infection); in the successive periods, temperature and availability of water are fundamental to produce sporangia (secondary infection) [54]. Since the mean temperature registered during the three considered growing seasons were not so diverse, the different rainfall amount might explain the significant difference in the disease symptoms found both on leaves and grapes in 2016 (more severe) than in 2017 and 2018 (less severe).

Exploring PM resistance behaviour over years, in 2016 the rainfall was high between May and June, determining the timing of ascospore release, followed by a peak of humidity in June allowing fungus growth (Figure S2). Under these conditions the more susceptible conditions in 2016 can be explained compared to less humid periods in 2017 and 2018, that probably limit the development of the disease. As stated in [55], the ideal conditions for the growth of PM were the temperature from 20°C to 28°C with 80%–90% relative humidity. In 2017 and 2018, the mean temperature was higher with peaks near 35°C in July and August, in contrast to 2016 where average temperatures were lower (Figure S3). As reported by [38,56], high temperatures could inhibit spore germination and slow down the growth of the fungus, until the death of the spores when temperatures reached 40°C. 

Pairwise correlation analyses reveal that, in a 3-year average, field resistance before veraison significantly correlates with resistance before harvest (*p* < 0.001, Figure S4). Especially for DM symptoms, correlation reached values of *ρ* = 0.746 and 0.824 for leaves and grapes, respectively. This correlation was slightly lower in PM symptoms with respectively *ρ* = 0.579 and 0.571.

To date, very few articles are available to be compared to the data presented here evaluated in such a range of genotypes. Some research dealt with the evaluation of hybrids in terms of performance (e.g., [57]) and most studies on QTL analysis of disease resistance against DM and PM resorted to use regularly leaf disc experiments (in vitro assay). For DM assessment in untreated fields, our study could be compared with the recent work of [58] where 28 promising hybrids were screened with a leaf discs assay and evaluated for foliar and cluster downy mildew resistance in an untreated field trial over three successive years. The common genotypes were only height, namely, ‘Aromera’, ‘Bronner,’ ‘Cabernet Cortis’, ‘Fanny’, ‘Leon Millot’, ‘Muscaris’, ‘Nero’ and ‘Pölöskei Muskotaly’. ‘Bronner’ resulted as the most resistant genotype at both leaves and cluster level in both works. Two additional resistant genotypes were ‘Muscaris’, that in the previous work appeared highly resistant as well, and ‘Leon Millot’. Surprisingly, ‘Leon Millot’ had a very high level of resistance in three successive years for both leaves and bunches, while in [58] the level of resistance was medium high for the leaves, and moderate for the grape cluster. Considering the remaining common genotypes, ‘Aromera’ shows a lower level of resistance in both organs, ‘Cabernet Cortis’ and ‘Fanny’ have higher levels of resistance in the leaves. In ‘Nero’ we noticed a lower level for leaves, but a higher level for bunches. Finally, ‘Pölöskei Muskotaly’ has shown the same mean behaviour for both. Another comparison for DM assessment using OIV 452 descriptor (leaves) in field exposed to natural pressure of pathogen was the work by [59]: The common genotypes were ‘Lela’, ‘Liza’, ‘Mila’, ‘Petra’, ‘Cerason’, ‘Seibel 13666′ and ‘Seyve Villard 12375′. ’Lela’, ‘Mila’, ‘Seibel 13666′ and ‘Seyve Villard 12375′ showed similar data with slight differences in the mean scores (OIV 452). Very different, on the contrary, were ‘Liza’ and ‘Petra’ that showed clearly higher values. Totally different was ‘Cerason’ that resulted susceptible, compared to a medium resistance in the Czech fields. In another work by [60], the only genotype in common was ‘Leon Millot’ where it showed once again a high level of field resistance, but the assessments were evaluated for several years carrying one or two fungicide treatments and the assessment of mildew damage was evaluated with a five grade scale taking into account leaves, shoots and berries. In [61], evaluations for DM and PM resistance were performed under field conditions in Hungary. The common genotypes were five, ‘Amadeus’, ‘Korai Bibor’, ‘Orpheus’, ‘Viktoria Gyöngye’ and ‘Duna Gyöngye’. Since these last two genotypes resulted not to be TTT in our work, they could not be compared. For DM resistance, the remaining genotypes displayed the same resistance levels for foliar (‘Korai Bibor’ and ‘Orpheus’) and bunches (‘Amadeus’ and ‘Orpheus’). In ‘Amadeus’ foliar resistance was detected at a lower degree, the same happened for bunches in ‘Korai Bibor’. For PM resistance, ‘Orpheus’ had similar result whereas ‘Korai Bibor’ showed definitely lower resistance levels in bunches whereas ‘Amadeus’ showed a higher resistance levels in both organs, especially in leaves.

### 2.4. Comparison between Mildew Resistance in Untreated Field and the Arrangement of Rpv or Run/Ren Loci

**Figure 4:**
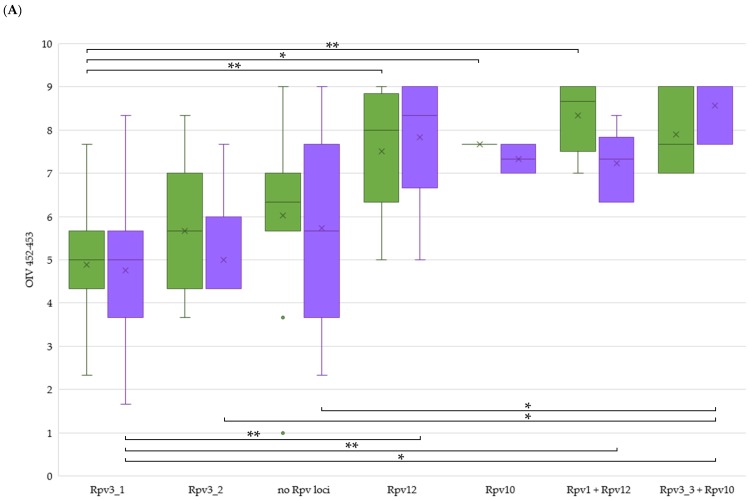

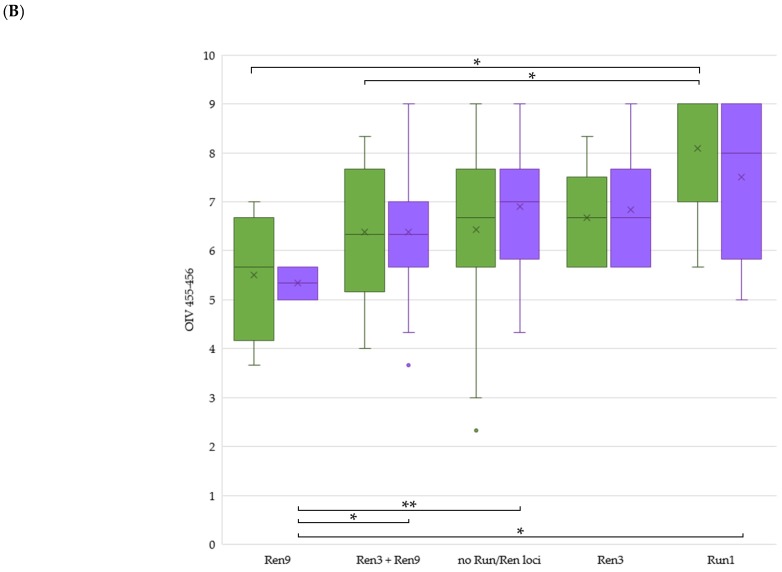
Evaluation of field resistance to downy (**A**) and powdery (**B**) mildew on leaves (green) and bunches (violet) on clustered accessions according to the presence of resistance (*R*) loci. Per each accession, OIV scores were averaged by 3-years. Per each year, the lowest score between evaluations was chosen. Box plots show medians by middle line and means by x. *: *p* < 0.05; **: *p* < 0.01.

Merging the field resistance data with the presence of single or stacked *R*-loci, the contribution of a particular genetic arrangement to the level of resistance in the field could be analysed. We excluded resistance data and genetic arrangements represented by a single genotype, since they were not informative during ANOVA testing. Concerning DM resistance (Figure 4A), the single *Rpv10* and *Rpv12* showed a significantly higher degree of 3-year field resistance on leaves compared to genotypes with the single *Rpv3-1*. About bunches, this higher rank is confirmed only for the comparison between *Rpv12* and *Rpv3-1.* The stacked *Rpv1* + *Rpv12* genotypes presented a significantly higher resistance than the single *Rpv3-1* genotypes both at leaf and bunch level. Regarding bunches only, DM resistance was significantly higher in genotypes containing *Rpv10* stacked with *Rpv3-3* compared to genotypes with the single *Rpv3-1* and *Rpv3-2*. No other *R*-loci (single or stacked) presented significant differences. Interestingly, genotypes without any detected *Rpv* locus revealed themselves not to be significantly more susceptible except for the stacked *Rpv3-3* + *Rpv10* genotypes at bunch level. Our findings highlight that as single players the resistance genes coming from *V. amurensis* (*Rpv10* and *Rpv12*) guarantee a strong barrier against the pathogen, although they do not seem to benefit from stacking other studied *Rpv* loci. These findings are in contrast with previous results mainly on leaves and can be attributed to differences in the genetic backgrounds. In fact, as reported by [22], a higher resistance behaviour could be pointed out in the seedlings containing both resistance genes coming from the two parental lines ‘Regent’ (*Rpv3*) and ‘VHR 3082-1-42′ (*Rpv1*). In [39], the authors describe *Rpv12* having an additive effect with *Rpv3* to protect grapevines against natural infections. Also in [40], the F1 sub-population which contains the *Rpv3* as well as the *Rpv10* locus showed a significantly higher degree of resistance, indicating additive effects by pyramiding of *R*-loci. 

Regarding PM (Figure 4B), *Run1* genotypes showed a significantly higher resistance compared to single *Ren9* and stacked *Ren3* + *Ren9* genotypes at leaf level. On bunches, both *Run1* and stacked *Ren3* + *Ren9* genotypes displayed a significantly higher resistance compared to the single *Ren9* genotypes. In the case of the single *Ren9* locus, bunch resistance levels were significantly decreased even compared to accessions without any studied *R*-locus, indicating a strong lack of resistance efficiency of this single locus. In fact, resistance QTL can sometimes only be detected under certain environmental conditions (soil, climate, pathogen population), or in specific genetic backgrounds. Thus, stable QTL are highly sought after for their applicability in breeding [62]. Our findings show that *Run1* confers a very strong resistance which derived from *Muscadinia* compared to the other loci derived from *Vitis* spp. Unlike *Rpv* loci in our study, *Ren* loci comparison revealed that pyramiding (i.e., *Ren3* + *Ren9*) allows the reinforcement of PM resistance at bunch level, the most delicate and relevant organ of the grapevine. In general, there is rather limited knowledge about the resistance mechanisms encoded in the various *Ren/Run* loci [63]. In the case of the flanked *Ren3/Ren9* loci, they exhibit a hypersensitive response to *E. necator* evident at 5 days post inoculation. PM resistance of ‘Regent’ relies on a “post-invasion” mechanism that restricts pathogen development and finally impairs the formation of conidia [46].

While observing seven genotypes without any known *R*-loci (‘Amadeus’, ‘Bruskam’, ‘Leon Millot’, ‘Orpheus’, ‘IV035′ and ‘IV063′), two of them, ‘Bruskam’ and ‘Leon Millot’, exhibited a high level of OIV scores (7 < average OIV descriptor ≤ 9) both in leaves and clusters for DM and in clusters for PM, while the foliar resistance against PM was < 7. In addition, ‘Amadeus’ had a high resistance against PM and against DM only in bunches, while the foliar resistance against DM was < 7. For the remaining three genotypes (‘Orpheus’, ‘IV063′ and ‘Semonell’) the levels of the average OIV score were medium (5 < average OIV descriptor ≤ 7) on both organs for both mildews. ‘IV035′ shows a medium level, except for higher level in bunches against PM. If we consider the resistance to a single disease, ten genotypes were lacking *Rpv* and 34 were missing *Run/Ren* loci. Fifty percent of the genotypes without any screenable *Rpv* locus disclosed a ≥ 5 average OIV score, whereas the percentage of genotypes without feasible *Run/Ren* loci with a medium high resistance level (> 5 average OIV score) was even higher (94%). In general, genotypes showing no analyzed *R*-loci may indicate the possible presence of minor loci (in the case of low-mid phenotypic scores) or even the discovery of novel, not yet identified, *R*-loci (in case of high phenotypic scores). These genotypes should be considered as precious resources in the perspective of pyramiding towards durable resistance. Indeed, while MAS facilitates major resistance gene pyramiding, it appears inapplicable to capture small-effect loci. However, field observations suggest that putative minor factors are involved in the expression of weaker but significant effects that can enhance the protection conferred by major genes and improve even more the stability [27]. Thus, in parallel to pursuing QTL studies, the implementation of genome-wide association studies and genomic selection in grapevine breeding [64] will certainly bring new opportunities to combine both major *R* genes and quantitative resistance and thus construct new varieties with highly durable resistance. The durability of grapevine QTL pyramids is now under evaluation. Durable disease resistance is a complex phenomenon having no one genetic or molecular basis, and the success of an integrated strategy can be judged only in retrospect. Finally, we suggest that the combination of disease resistance genes or QTLs with other approaches for pathogen control (pesticide *ad hoc* application, farming practices) may be a relevant management strategy to slow down the evolution of virulent pathogen genotypes.

## 3. Materials and Methods

### 3.1. Genetic Material and DNA Isolation

The plant material consisted of 102 grapevine accessions divided into 65 breeding lines and 37 progeny individuals. The 65 breeding lines originated from breeding programs in various European institutions located in Germany, France, Austria, Hungary, Czech Republic, Russia and Switzerland. The remaining 37 genotypes were progeny individuals derived from different crosses made by a private breeder (InnoVitis, Marlengo (BZ), Italy) with the aim to introgress disease resistance traits into good quality *V. vinifera* backgrounds. The whole genetic material (Table S3)—in this study referred as VITISANA collection—was cultivated in an unsprayed vineyard located in a private winery in Marlengo (BZ, Italy) (N 46.670938, E 11.131313, 401 m a.s.l.). Each genotype was present at least in triplicate, managed since 2010 using a Guyot training system with a planting density of 2 m × 0.8 m in terraced fields.

Genomic DNA was extracted from young frozen leaves (0.08 g) using DNeasy Plant Kit (Qiagen, Hilden, Germany) in accordance with manufacturer’s instructions. After extraction, DNA was quantified using a NanoDrop 8000 Spectrophotometer (Thermo Fisher Scientific, Waltham, MA, USA) and the DNA concentration was normalized to 10 ng/µL.

### 3.2. Trueness-to-Type Analysis

All the hybrids were genetically characterized with the nine reference SSRs, internationally approved for the genetic fingerprinting and the consequent identification of grapevine varieties according to the GenRes081 and GrapeGen06 EU project [65,66]. The amplifications were performed in a GeneAmp 9700 thermal cycler (Thermo Fisher Scientific, Waltham, MA, USA) in a 10 µL final volume using Qiagen Multiplex Kit (Qiagen, Hilden, Germany) according to the manufacturer’s instructions. The nine SSR primer pairs were divided into three multiplex PCRs using different fluorescent dyes as reported in the Table S4. The following PCR profile was applied: Precycle 15 min at 95°C, 40 cycles of 40 sec denaturation at 95°C, 90 sec annealing at 55°C and 90 sec extension at 72°C, final extension of 30 min at 60°C. Capillary electrophoresis was carried out in an ABI 3130xl Genetic Analyzer (Life Technologies, Foster City, CA, USA) and the fragments (alleles) were sized with GeneMapper v4.0 in binning mode, using GeneScan 500LIZ size standard as an internal ladder (Life Technologies, Foster City, CA, USA). The trueness-to-type (TTT) was verified against the VIVC database [9].

### 3.3. R-Loci Analysis 

Following the “all-vs-all” approach reported by [67,68], all *Vitis* hybrids were examined at the 11 actually screenable and reliable *R*-loci: Five associated with DM resistance (*Rpv1*, *Rpv3*, *Rpv10*, *Rpv12*, *Rpv14*) and six associated with PM resistance (*Run1*, *Run2*, *Ren1*, *Ren2*, *Ren3* and *Ren9*). The symbol of the resistance genes, the name of the causal agents of the diseases (traits), the resistance-related markers, the alleles associated with the resistance and the genotypes of origin are reported in Table 2. PCR amplifications were carried out according to the protocols optimized by [67]. Herein absence of *R*-loci (no *R*-loci) stands solely for the analysed loci and leaves the possibility of known loci without exploitable markers or new /unknown loci.

### 3.4. DM and PM Symptom Phenotyping in Field

Symptoms of DM and PM natural infections were assessed on the 102 accessions in untreated plots over three growing seasons (2016, 2017 and 2018). The scores were collected twice during each season, before veraison and before harvest. The OIV descriptors 452 and 453 were used for DM and OIV descriptors 455 and 456 for PM symptom assessments, on leaf laminas and clusters respectively. The organs were visually inspected by the same two trained evaluators and the scoring was carried out in the same two days for all hybrids. The maximum level of symptom expression (lowest OIV score) between the two times was considered within each season. Weather conditions including average rainfall, temperature and relative humidity were tracked from April to September in the local weather website (http://meteo.provincia.bz.it/dati-storici.asp, indicating Merano for the location). The weather station is located at about 2.0 km distance from the vineyard. 

### 3.5. Statistical Analysis

Based on the 9 universal SSR markers, a genetic distance analysis was attempted by means of PAST v.3.14 software [69], applying Neighbour-joining with Euclidean distance. Statistical analyses on field resistance evaluations were performed using SPSS Statistics v24 (IBM). The year effect was analysed by pairwise comparing the mean values of the lowest assessments for each year with Friedman’s two-way Analysis of Variance (ANOVA) by ranks adjusted by Bonferroni correction (*p* < 0.001). Relationships between assessments before veraison and before harvest were calculated using Spearman’s bivariate correlation tests (*p* < 0.001) on a 3-year mean. One-Way ANOVA together with Post-Hoc Games–Howell test (*p* < 0.05) was used for pairwise comparisons of groups having different *R*-loci.

## 4. Conclusions

The analysis of all reliably applicable *R*-loci in the VITISANA collection—the so-called “all-vs-all” approach—turned out to be crucial to detect the presence of *Rpv* and *Run/Ren* loci in a definitive way, regardless of the supposed resistance donor(s) according to the historical pedigree information. The list of reliable markers is limited compared to the entire number of QTLs discovered and published until now on grapevine. For this reason, during selection activities, it is important to include also field evaluations in order to recover (mid)-resistant genotypes that would otherwise be discarded during sole marker analysis. In this regard, our survey allowed the discovery of several new sources of disease resistance useful for next coming QTL identifications, which are valuable and exploitable for MAB purposes. In addition, this study highlights *Ren* loci stacking to be effective on PM resistance especially at bunch level. At the same time, in the case of the presence of a prominent *Rpv* locus, pyramiding does not necessarily mean an increase of DM resistance level, but potentially provides a second barrier in case of pathogens overcoming the first one. As for traditional varieties, resistant varieties should be adapted to particular *terroirs*. Here there is still a huge lack of experience, which would enable possible adaptations to be identified in order to boost the ongoing diffusion of these novel varieties requested for sustainability issues.

## Figures and Tables

**Table 1:** Characterization of resistance (*R*) loci in each studied accession based on the “all-vs-all” approach: The occurrence of the *R*-locus is highlighted in red, while its absence is represented by an empty cell.

Accession Name	InnoVitis Code	TTT	Rpv1	Rpv3-1 299-279	Rpv3-2 null-297	Rpv3-3 null-271	Rpv3 321-312	Rpv3 361-299	Rpv3 299-314	Rpv3 null-287	Rpv10	Rpv12	Run1	Ren1	Ren3	Ren9
1/04/09	IV130	not available													Ren3	Ren9
1/1/06	IV109	not available		Rpv3-1 299-279											Ren3	
1/16/04	IV090	not available		Rpv3-1 299-279												Ren9
1/24/02	IV091	not available		Rpv3-1 299-279												Ren9
1/24/09	IV097	not available		Rpv3-1 299-279											Ren3	
1/26/4	IV132	not available		Rpv3-1 299-279											Ren3	Ren9
1/39/11	IV119	not available		Rpv3-1 299-279											Ren3	Ren9
1/41/07	IV104	not available		Rpv3-1 299-279											Ren3	
1/43/03	IV101	not available		Rpv3-1 299-279												
1/5/06	IV112	not available			Rpv3-2 null-297										Ren3	Ren9
2/01/04	IV102	not available		Rpv3-1 299-279											Ren3	
2/04/01	IV131	not available			Rpv3-2 null-297										Ren3	Ren9
2/04/06	IV108	not available		Rpv3-1 299-279												
2/16/02	IV117	not available		Rpv3-1 299-279											Ren3	Ren9
2/21/06	IV088	not available		Rpv3-1 299-279												
2/22/07	IV078	not available		Rpv3-1 299-279											Ren3	Ren9
2/23/04	IV120	not available		Rpv3-1 299-279												
2/27/04	IV074	not available		Rpv3-1 299-279											Ren3	Ren9
2/27/06	IV077	not available			Rpv3-2 null-297										Ren3	Ren9
2/31/10	IV098	not available		Rpv3-1 299-279											Ren3	Ren9
2/41/01	IV086	not available		Rpv3-1 299-279											Ren3	Ren9
3/1/06	IV076	not available		Rpv3-1 299-279												
3/14/10	IV096	not available		Rpv3-1 299-279												Ren9
3/18/05	IV085	not available		Rpv3-1 299-279											Ren3	Ren9
3/22/09	IV133	not available		Rpv3-1 299-279												
3/23/05	IV075	not available		Rpv3-1 299-279												
3/24/09	IV125	not available		Rpv3-1 299-279												
3/25/10	IV083	not available		Rpv3-1 299-279												
3/33/08	IV114	not available		Rpv3-1 299-279											Ren3	Ren9
3/34/08	IV107	not available		Rpv3-1 299-279											Ren3	Ren9
3/35/09	IV106	not available		Rpv3-1 299-279											Ren3	Ren9
3/37/02	IV072	not available		Rpv3-1 299-279											Ren3	Ren9
3/45/09	IV134	not available		Rpv3-1 299-279												
4/35/01	IV129	not available													Ren3	Ren9
4/45/01	IV126	not available		Rpv3-1 299-279												Ren9
4/46/04	IV100	not available		Rpv3-1 299-279												
4/5/08	IV110	not available		Rpv3-1 299-279												
ALFA	IV042	putative		Rpv3-1 299-279												
AMADEUS	IV184	putative														
*V. AMURENSIS CHINENSIS*	IV055	putative									Rpv10					
AROMERA	IV004	putative		Rpv3-1 299-279											Ren3	Ren9
BRONNER	IV001	yes				Rpv3-3 null-271					Rpv10				Ren3	Ren9
BRUSKAM	IV038	putative														
CABERNET CORTIS	IV051	putative				Rpv3-3 null-271					Rpv10				Ren3	Ren9
CERASON	IV028	putative		Rpv3-1 299-279											Ren3	Ren9
(COARNA N. × PIERELLE) × SV 20366	IV037	putative		Rpv3-1 299-279											Ren3	Ren9
DONAURIESLING	IV185	putative		Rpv3-1 299-279											Ren3	Ren9
DUNA GYÖNGYE	IV032	no		Rpv3-1 299-279											Ren3	
ESTHER	IV013	yes		Rpv3-1 299-279											Ren3	Ren9
FANNY	IV011	yes						Rpv3 361-299							Ren3	
GEORG	IV163	putative		Rpv3-1 299-279											Ren3	Ren9
GM 6495-3	IV213	yes				Rpv3-3 null-271					Rpv10					
InnoVitis 029	IV029	not available										Rpv12				Ren9
InnoVitis 035	IV035	not available														
InnoVitis 039	IV039	not available		Rpv3-1 299-279											Ren3	Ren9
InnoVitis 043	IV043	not available	Rpv1									Rpv12	Run1			
InnoVitis 045	IV045	not available										Rpv12			Ren3	Ren9
InnoVitis 046	IV046	not available		Rpv3-1 299-279								Rpv12			Ren3	Ren9
InnoVitis 061	IV061	not available										Rpv12	Run1			
InnoVitis 062	IV062	not available		Rpv3-1 299-279											Ren3	Ren9
InnoVitis 063	IV063	not available														
InnoVitis 064	IV064	not available	Rpv1									Rpv12	Run1			
InnoVitis 065	IV065	not available	Rpv1										Run1			
InnoVitis 066	IV066	not available	Rpv1									Rpv12	Run1			
InnoVitis 067	IV067	not available	Rpv1									Rpv12	Run1			
InnoVitis 068	IV068	not available	Rpv1									Rpv12	Run1			
InnoVitis 069	IV069	not available										Rpv12			Ren3	Ren9
InnoVitis 070	IV070	not available	Rpv1									Rpv12	Run1			
InnoVitis 183	IV183	not available														
KATHARINA	IV164	yes		Rpv3-1 299-279	Rpv3-2 null-297											
KISHMISH VATKANA	IV034	yes												Ren1		
KORAI BIBOR	IV186	putative														Ren9
KUNLEANY	IV030	yes										Rpv12				
LELA	IV187	putative										Rpv12				
LEON MILLOT	IV021	yes														
LIZA	IV188	putative										Rpv12				
LU 1	IV040	putative		Rpv3-1 299-279							Rpv10					Ren9
LU 2	IV036	putative									Rpv10				Ren3	Ren9
MILA	IV189	putative										Rpv12				
MUSCARIS	IV008	putative									Rpv10				Ren3	Ren9
MUSCAT BLEU	IV010	yes		Rpv3-1 299-279			Rpv3 321-312									
MW 1	IV027	putative		Rpv3-1 299-279	Rpv3-2 null-297										Ren3	Ren9
NERO	IV016	yes		Rpv3-1 299-279											Ren3	Ren9
ODYSSEUS	IV190	no										Rpv12				
ORPHEUS	IV191	yes														
PALATINA	IV009	yes		Rpv3-1 299-279												
PETRA	IV195	yes										Rpv12				
PHILIPP	IV161	yes		Rpv3-1 299-279											Ren3	Ren9
PÖLÖSKEI MUSKOTALY	IV012	yes		Rpv3-1 299-279											Ren3	
SEIBEL 13666	IV053	yes		Rpv3-1 299-279											Ren3	Ren9
SEMONELL	IV041	not available														
SEYVE VILLARD 12375	IV047	yes		Rpv3-1 299-279				Rpv3 361-299							Ren3	Ren9
SEYVE VILLARD 12-481	IV054	putative		Rpv3-1 299-279												Ren9
SK 76-1/4	IV196	putative			Rpv3-2 null-297										Ren3	Ren9
SK 77-4/5	IV197	putative													Ren3	Ren9
SOLIRA	IV023	putative		Rpv3-1 299-279											Ren3	Ren9
SOPHIE	IV162	yes		Rpv3-1 299-279											Ren3	
SOUVIGNIER GRIS	IV044	putative			Rpv3-2 null-297										Ren3	Ren9
VIKTORIA GYÖNGYE	IV031	no		Rpv3-1 299-279											Ren3	
VINERA	IV017	putative			Rpv3-2 null-297											
VINORÈ	IV025	putative		Rpv3-1 299-279											Ren3	Ren9
ZARYA SEVERA	IV165	no				Rpv3-3 null-271					Rpv10					

**Table 2:** Resistance (*R*) loci against *Plasmopara viticola* and *Erysiphe necator* with chromosome, used associated markers and their relative resistance alleles/haplotype, and used reference genotypes.

Symbol	Resistance Trait	Chromosome	Associated Marker	Resistant Alleles/Haplotype	Reference Genotype
*Rpv1*	*Plasmopara viticola*	12	VMC1g3.2	122	VRH30-82-1-42
			VMC8g09	160	
*Rpv3-1*	*Plasmopara viticola*	18	UDV305	299	Villard Blanc
(= *Rpv3* ^299-279^)			UDV737	279	
*Rpv3-2*			UDV305	null	Seyval
(= *Rpv3* ^null-297^)			UDV737	297	
*Rpv3* ^321-312^			UDV305	321	Chancellor
			UDV737	312	
*Rpv3-3*(= *Rpv3*^null-271)^			UDV305	null	Seyval
			UDV737	271	
*Rpv3* ^361-299^			UDV305	361	Villard Blanc
			UDV737	299	
*Rpv3* ^299-314^			UDV305	299	Couderc 13
			UDV737	314	
*Rpv3* ^null-287^			UDV305	null	Chancellor
			UDV737	287	
*Rpv10*	*Plasmopara viticola*	9	GF09-44	230	Severnyi
			GF09-46	416	
			GF09-47	299	
*Rpv12*	*Plasmopara viticola*	14	UDV340	197	Zarya Severa
			UDV345	236	
			UDV 360	227	
*Rpv14*	*Plasmopara viticola*	5	GF05-13	228	Börner
			UDV111	114	
*Run1*	*Erysiphe necator*	12	VMC4f3.1	186	VRH3082-1-42
			VMC8g9	160	
			Sc34-8	216	
			Sc35-2	238	
*Run2.1*	*Erysiphe necator*	18	VMC7f2	193	Magnolia
			UDV108	202	
*Run2.2*	*Erysiphe necator*	18	VMC7f2	195	Trayshed
			UDV108	220	
*Ren1*	*Erysiphe necator*	13	Sc47-18	249	Kishmish vatkana
			SC08_0071_014	143	
*Ren3*	*Erysiphe necator*	15	GF15-42	199	Regent
			ScORGF15-02	242	
*Ren9*	*Erysiphe necator*	15	CenGen6	287	Regent

## Supplementary Materials

Supplementary materials can be found at https://www.mdpi.com/1422-0067/20/14/3526/s1.
