# Supplementary material for: R-Loci Arrangement Versus Downy and Powdery Mildew Resistance Level: A Vitis Hybrid Survey
Source: Int J Mol Sci. 2019 Jul 18;20(14):3526. doi: 10.3390/ijms20143526 (PMC6679420; doi:10.3390/ijms20143526)
Supplement: Supplementary file 1 [file ijms-20-03526-s001.zip › Figures_S1-S4.docx]

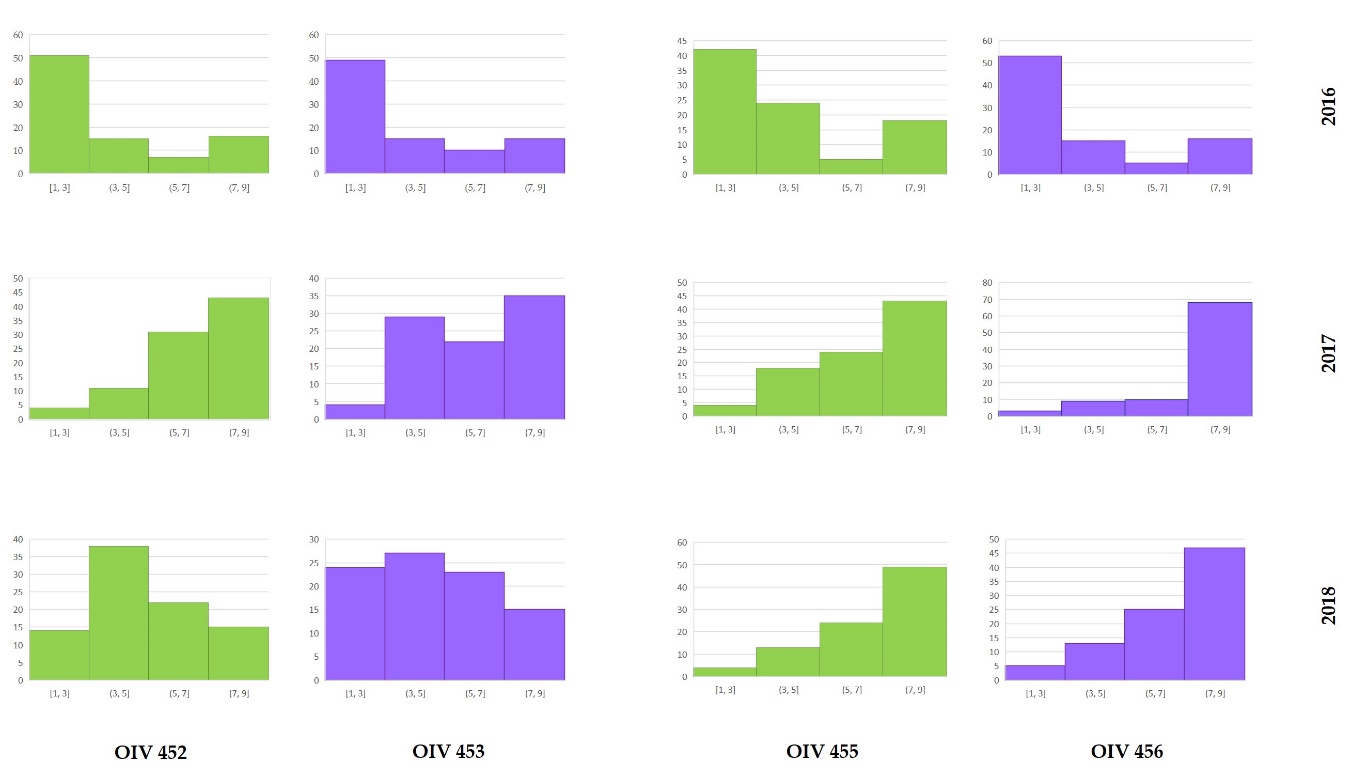


**Figure S1.** Histograms showing the distribution of OIV scores corresponding to downy (A) and powdery (B) mildew resistance level in leaves (green) and bunches (violet) in 2016, 2017 and 2018. Per each year the lowest score between evaluations was chosen.


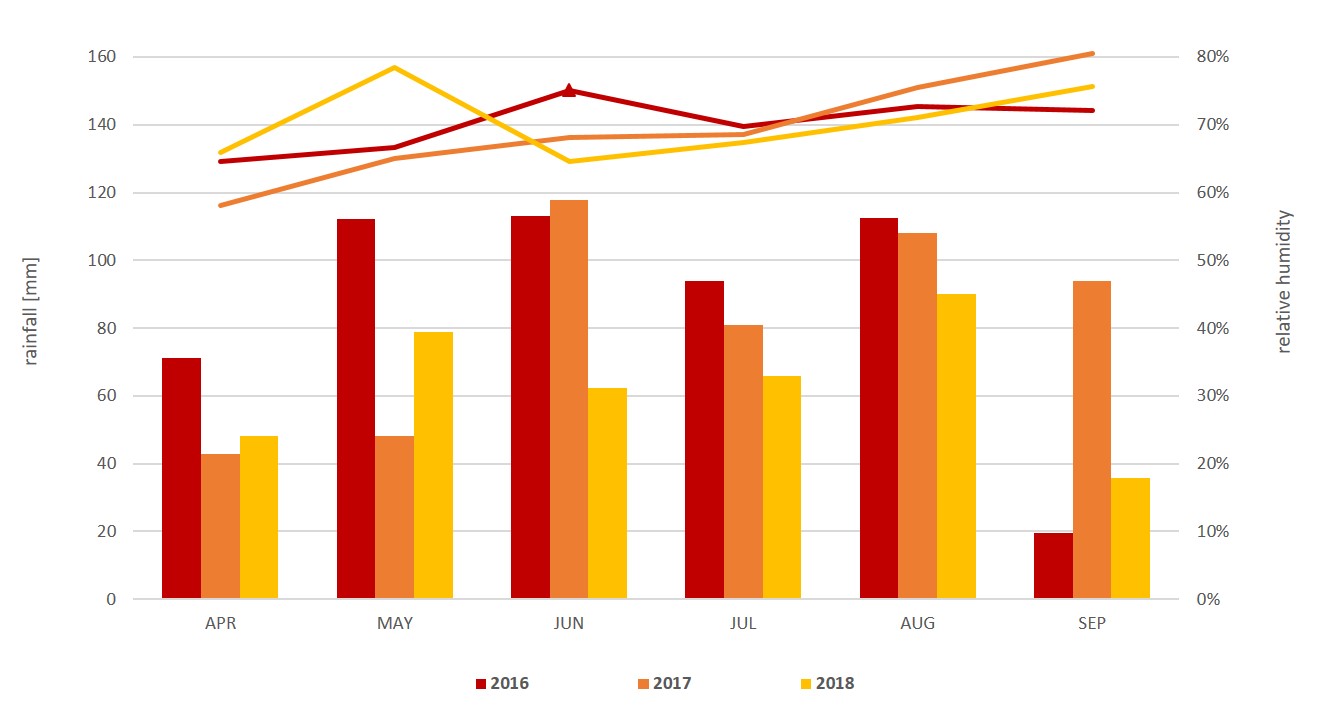


**Figure S2.** Weather conditions between April and September of the seasons 2016 (red), 2017 (orange) and 2018 (yellow). Cumulative quantities of rainfall per month are represented by bars in mm, monthly averaged relative humidity is represented by lines in percent. The peak of relative humidity in 2016 is marked by a triangle.


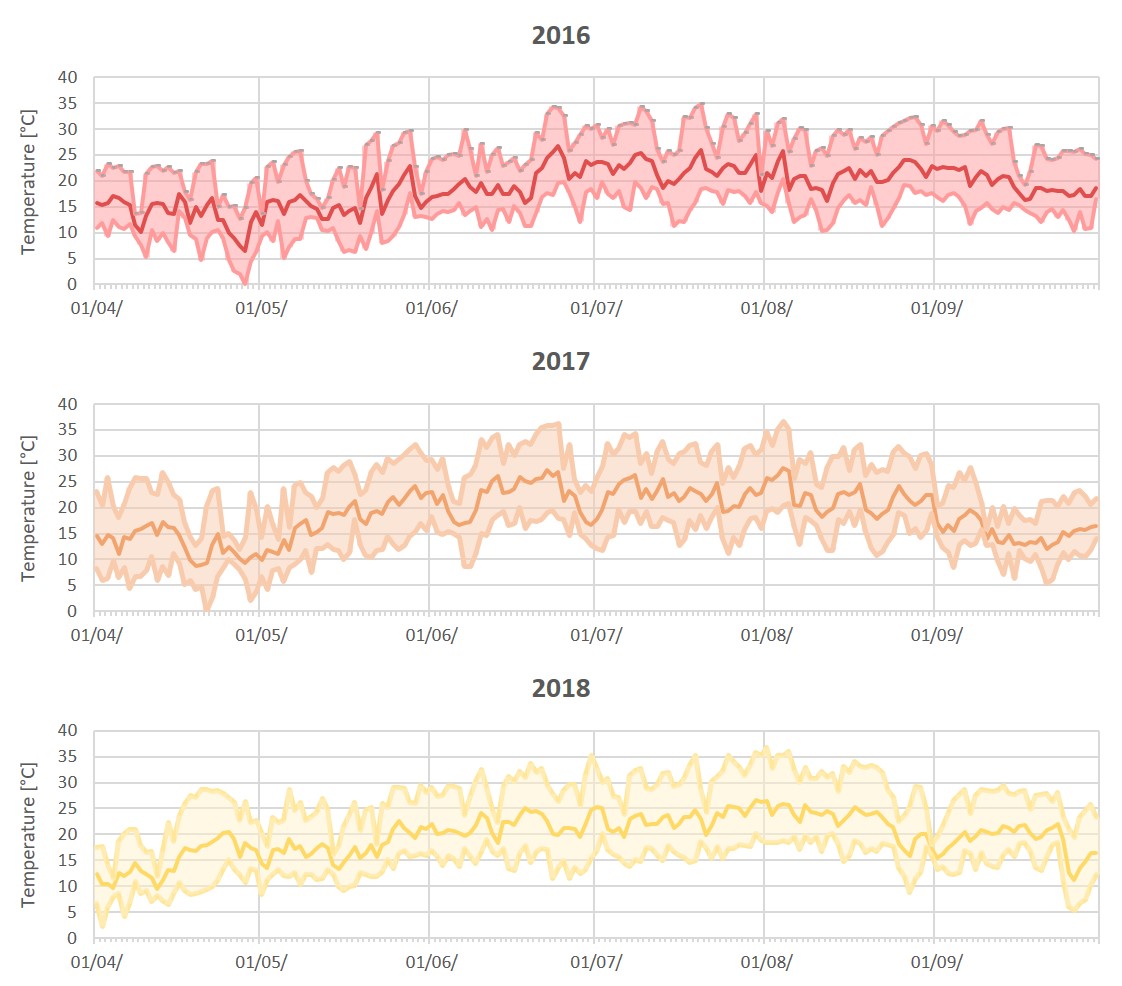


**Figure S3.** Temperature profiles from April 1^st^ to September 30^th^ of the seasons 2016, 2017 and 2018. Upper and lower lines represent maximal and minimal daily temperatures, respectively. Middle line represents daily average temperature.


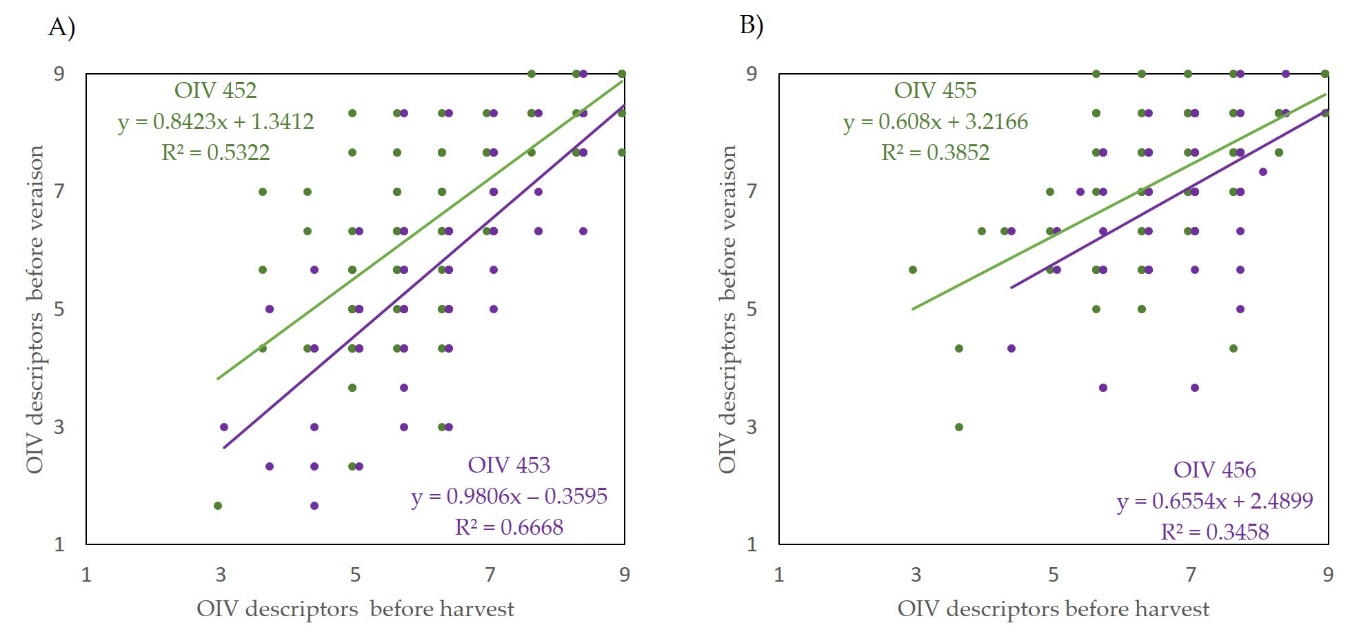


**Figure S4.** Linear regression between field resistance before veraison and before harvest to downy (A) and powdery (B) mildew on 89 accessions in a 3-year average. Leaf (green) and bunch (violet) resistance was evaluated.
